# Genome-Wide Identification of Specific Genetic Loci Common to Sheep and Goat

**DOI:** 10.3390/biom14060638

**Published:** 2024-05-29

**Authors:** Zuoxiang Liang, Xiaoyu Yue, Yangxiu Liu, Mengyan Ye, Ling Zhong, Yue Luan, Qin Wang

**Affiliations:** 1 State Key Laboratory of Animal Biotech Breeding, National Engineering Laboratory for Animal Breeding, Laboratory of Animal Genetics, Breeding and Reproduction, Ministry of Agriculture and Rural Affairs, College of Animal Science and Technology, China Agricultural University, Beijing 100193, China; zliang@umn.edu (Z.L.); b20233040387@cau.edu.cn (X.Y.); 18813039611@163.com (Y.L.); mengyanye@cau.edu.cn (M.Y.); sy20213040767@cau.edu.cn (L.Z.); luanyue@cau.edu.cn (Y.L.); 2 Department of Animal Science, University of Minnesota, Saint Paul, MN 55108, USA

**Keywords:** SGCSSs, species identification, PCA, MNSAs, D values

## Abstract

Sheep and goat may become carriers of some zoonotic diseases. They are important livestock and experimental model animals for human beings. The fast and accurate identification of genetic materials originating from sheep and goat can prevent and inhibit the spread of some zoonotic diseases, monitor market product quality, and maintain the stability of animal husbandry and food industries. This study proposed a methodology for identifying sheep and goat common specific sites from a genome-wide perspective. A total of 150 specific sites were selected from three data sources, including the coding sequences of single copy genes from nine species (sheep, goat, cow, pig, dog, horse, human, mouse, and chicken), the dbSNPs for these species, and human 100-way alignment data. These 150 sites exhibited low intraspecific heterogeneity in the resequencing data of 1450 samples from five species (sheep, goat, cow, pig, and chicken) and high interspecific divergence in the human 100-way alignment data after quality control. The results were proven to be reliable at the data level. Using the process proposed in this study, specific sites of other species can be screened, and genome-level species identification can be performed using the screened sites.

## 1. Introduction

Sheep (*Ovis aries*) and goat (*Capra hircus*), as two popular livestock, are not only important sources of meat, pelt, wool fiber, and dairy for human consumption but also have been utilized for scientific research on genetics and disease models [1,2,3,4,5]. However, sheep and goat are also potential carriers of many zoonotic pathogens [6]. A famous example is bovine spongiform encephalopathy (BSE), one type of transmissible spongiform encephalitis (TSE), leads to brain damage or death [7]. It was reported that sheep and goat BSE can be transmitted more efficiently than cow BSE in transgenic mice with human prion protein (PrP) [8]. Another serious zoonosis, brucellosis, also could be transmitted from sheep or goat to humans [9]. This disease, which is caused by the genus Brucella, threatens public health in many parts of the world [10,11]. Therefore, detecting genetic materials originating from sheep and goat is important for both food and public health surveillance.

Over the past few decades, the mitochondrial DNA (mtDNA) is widely utilized for species identification [12]. mtDNA, a small DNA located in mitochondria, is characterized by maternal inheritance, a high mutation rate, a low recombination level, and a high level of interspecies divergences [13]. Haider et al. reported that the cytochrome c oxidase subunit I (*COI*) gene of mtDNA, along with the polymerase chain reaction (PCR)–restriction fragment length polymorphism (RFLP) technique, can be used to efficiently distinguish eight species, including cow, chicken, turkey, sheep, pig, buffalo, camel, and donkey [14]. Recently, Jahura et al. found that two conserved fragments of the mitochondrial cytochrome b gene were able to accurately distinguish products originating from sheep and goat [15]. Although some successful examples exist, the application of mtDNA is limited due to its relatively low resolution and inability for quantification.

As next-generation sequencing (NGS) technologies become more and more popular, high-quality genome assemblies and accurate genome annotations are rapidly accumulating for various organisms, as well as for whole-genome resequencing data [16,17,18,19,20]. An increasing number of studies prefer to identify molecular markers in nuclear genomes for species identification due to the limitation of mtDNA. Although the data volumes increase over many orders of magnitude, requiring more effort in data analysis, the application of nuclear genomes still provides the following advantages: (1) It provides plenty of regions featuring high intraspecific concordance and interspecific divergences. For example, ultra-conserved elements (UCEs), known for their high conservation, exhibit superior performance to infer species phylogeny [21,22]. (2) It offers a collection of single copy genes. Single copy genes evolve slower than duplicated genes, which helps to increase the accuracy in sequence alignments [23,24,25]. Furthermore, compared to mtDNA that has high copy number variations in different individuals or tissues, the sites from single copy genes without any copy number variation are more suitable for quantitative analysis using real-time PCR [26,27]. (3) It provides high reliability at the data level. Huge numbers of single-nucleotide polymorphisms (SNPs) for more and more species can be used to verify whether the species-specific sites selected from single copy genes for species identification are credible or not. In addition, SNP genotyping is a very cost-effective way for species identification and exhibits highly accurate performance under modern techniques including real-time PCR and mass spectrum.

In this study, we developed a pipeline to identify highly confident sheep and goat common specific sites (SGCSSs) to distinguish the genetic materials derived from sheep–goat (sheep and goat) and seven other species. By doing this, high-quality assemblies and annotated genomic data as well as SNP data from nine species were used, including sheep, goat, cow (*Bos taurus*), pig (*Sus scrofa*), dog (*Canis lupus familiaris*), horse (*Equus caballus*), human (*Homo sapiens*), mouse (*Mus musculus*), and chicken (*Gallus gallus*) for comprehensive searching and filtering. Subsequently, we obtained 150 candidate SGCSSs.

Next, we performed a verification test using the resequencing data to investigate whether these particular sites belong only to sheep–goat or not. The resequencing data contained a total of 1450 samples from five species: chicken, pig, cow, sheep, and goat. In addition, we also analyzed the interspecific differences in SGCSS with high confidence. This study has two important meanings: (1) The first is accurate quantification. The use of 150 SGCSS to distinguish sheep and goat from other common mammals could reduce testing costs and increase efficiency. (2) A new screening strategy was proposed, in which multi-parameter screening was carried out step by step based on a large amount of data, and then specific loci were found to be common between sheep and goat but almost non-existent in other common mammals. This method of screening large amounts of data to obtain specific sites is novel and innovative.

## 2. Materials and Methods

### 2.1. Data Collection

Three data sources were involved in this study: (1) High-quality genomic data for sheep, goat, cow, pig, dog, horse, human, mouse, and chicken were downloaded from the Ensembl database (release 92, http://www.ensembl.org/). These included gene transfer format (GTF) files, dbSNP (vcf format) files, soft masked genomic sequences (genome versions in Supplementary Table S1), and coding sequences (CDS). (2) Human 100-way alignments were obtained from the UCSC database (http://genome.ucsc.edu/). (3) The SNP information from 1450 whole-genome resequencing (WGS) individuals were collected from published data and the data from our lab (data not released). This dataset contained five species: *Ovis aries* (sheep), *Capra hircus* (goat), *Bos taurus* (cow), *Sus scrofa* (pig), and *Gallus* (chicken). Since *Ovis canadensis* (bighorn sheep), *Ovis dalli* (dall sheep), *Ovis orientalis* (mouflon), and *Ovis vignei* (urial) are closely related to *Ovis aries* (sheep), all of them were assigned to sheep populations. Similarly, *Capra aegagrus* (bezoar) and *Capra hircus* (goat) were both assigned to goat populations; *Bos taurus* × *indicus* (cow) and *Bos taurus* (cow) were both assigned to cow populations; and *Sus cebifrons* (Visayan warty pig), *Sus barbatus* (bearded pig), *Sus celebensis* (Celebes wild boar), *Sus verrucosus* (Java warty pig), *Phacochoerus africanus* (common warthog), and *Sus scrofa* (pig) were assigned to pig populations.

In total, sheep populations contained 377 samples from more than 51 breeds, goat populations contained 217 samples, cow populations contained 34 samples, pig populations contained 366 samples from more than 46 breeds, and chicken populations contained 456 samples from 21 breeds. Additional details of the dataset are described in Supplementary Table S2.

### 2.2. Gene Family Clusters Construction

The TreeFam methodology was applied to define a gene family as a group of genes descended from a single gene in the recent common ancestor of all animals [28]. The TreeFam pipeline with same parameters from the previous sheep genome project was used to infer the orthologs among the nine species [29]. In detail, the perl script “bp_translate_seq.pl” in BioPerl v1.69 was first used to translate CDS to the corresponding peptide sequence [30]. Next, “all-against-all” BLASTP (E-value < 1 × 10^−7^) was performed to compute the similarities of all the protein sequences of the nice species. High-scoring segment pairs (HSPs) were then concatenated for each gene pair by SOLAR. When the region aligned to both genes exceeded 1/3 of the sequence length, a connection (edge) between the two nodes (genes) was added. Finally, the hcluster_sg algorithm (https://github.com/douglasgscofield/hcluster) was used to cluster the homologous genes into the corresponding gene families.

### 2.3. Sequence Alignment Processing

Based on the constructed gene family clusters, single copy orthologs were first extracted from species reference genomes. Next, multiple peptide sequence alignment (MPSA) for each ortholog was conducted using SATé v2.2.7 and MAFFT v7.408 [31,32]. To select high-quality MPSAs, Gblocks v0.91b (parameters: -t=p -b3=15 -b4=5 -b5=n) was applied to extract conserved domain sequences from each MPSA [33]. An MPSA, in which the length of the aligned segment was shorter than 100 amino acids or the proportion of gaps was more than 20% of the total alignments, was discarded. Finally, the remaining MPSAs were converted back to the format of multiple nucleotide sequence alignments (MNSAs).

### 2.4. Searching Candidate SGCSSs

**Figure 1:**
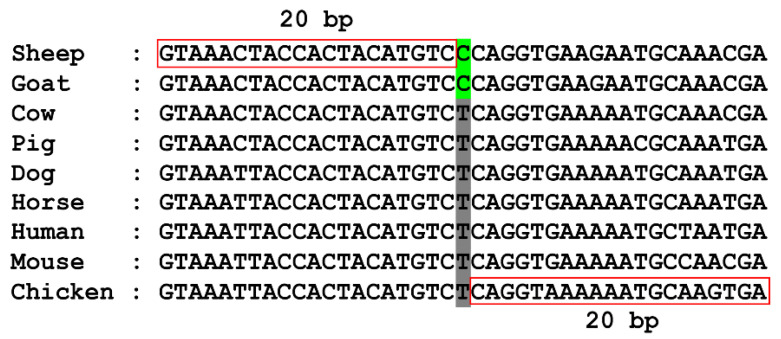
One MNSA window centered on candidate SGCSS (green) without gaps. The corresponding sites in the other seven species are shown in gray. The length of MNSA fragment surrounded by candidate SGCSS is 20 bp (red rectangle).

Next, we carried out a sliding window strategy with window size = 20 bp and step size = 1 bp on each MNSA to screen the candidate SGCSSs. The criteria for a candidate site extracted from MNSAs were as follows: (1) The nucleobase in sheep must be identical to that in goat. (2) The nucleobases in sheep and goat must be different from those in the other seven species (cow, pig, dog, horse, human, mouse, and chicken). (3) The nucleobases located at the corresponding genomic positions of the other seven species must be consistent. (4) The nucleotide relationship between the candidate SGCSS and its corresponding sites in the other seven species must not follow the principle of DNA base pair complementarity. For example, suppose that the nucleobase of one candidate SGCSS was “C” for sheep–goat, the corresponding nucleobases in the other seven species must be “A” or “T” but not “G” (Figure 1). (5) Gaps within 20 bp of the candidate SGCSS are not permitted.

The candidate SGCSS containing variants in the population genetic data for each species must be removed from the list obtained from the previous step. Therefore, we filtered out candidate SGCSSs that had SNPs using the dbSNP data for all nine species. The number of SNPs recording in the dbSNP database ranged from 5,019,522 for horse to 324,781,098 for human (Supplementary Table S3). The candidate SGCSSs, along with their flanking sequences, were then mapped back to the genome to obtain the exact genomic location of the SGCSSs and their corresponding sites in seven other species. To do this, probes containing the candidate SGCSS or the corresponding sites in other species were trimmed from each sequence of each MNSA (Supplementary Figure S1), with flanking sequences that ranged from 21 to 41 bp. For all nine species, only the unique match was retained for further analysis. Finally, we retained the results where both candidate SGCSSs and corresponding sites had no SNP records in dbSNP.

### 2.5. Scoring Candidate SGCSSs

In this step, we scored the candidate SGCSSs to select sites that have maximum discrimination power using the human 100-way alignment data. After filtering out SGCSSs that were not recorded in the human 100-way alignments, we then removed the locus with a frequency of missing sites more than 5%, and the species with a frequency of missing sites more than 10%. To do this, the items, including nucleobases and gaps, that occurred in the alignments for each site were divided into three groups. In detail, for each species, a nucleobase was assigned to group 1 if it was identical to that in the candidate SGCSS and to group 2 if it differed from that in the candidate SGCSS. The missing site was assigned to group 3. Therefore, the frequency for each group could be calculated as follows:

(1) $$p_{1i}=\frac{n_{1i}}{N}$$

(2) $$p_{2i}=\frac{n_{2i}}{N}$$

(3) $$p_{3i}=\frac{n_{3i}}{N}$$

where $n_{1i}$ is the counts of group 1 for the *i*th site, $n_{2i}$ is the counts of group 2 for the *i*th site, $n_{3i}$ is the counts of group 3 for the *i*th site, and *N* is the total number of species retained after quality control. We then utilized a $D_{i}$ value which is the difference between $p_{2i}$ and $p_{1i}$ to score *i*th candidate SGCSS. The formula could be expressed as follows:

(4) $$D_{i}=p_{2i}-p_{1i}$$

Finally, the top-ranked 150 SGCSSs with the highest *D* values were selected as the most informative SGCSSs.

### 2.6. The Specificity of SGCSS Analysis

To evaluate the performance of these 150 SGCSSs, we first merged these sites and their corresponding sites among other species from the human 100-way alignments into a pseudo-MNSA. We then conducted principal component analysis (PCA) and phylogeny construction on the MNSA. For the PCA, we first transformed the MNSA into an integer format, where each nucleobase in group 1 was given an integer value of 1, those in group 2 were set to −1, and those in group 3 were set to NA for each site. This step was implemented in scikit-learn v0.18 [34].We also built a maximum-likelihood phylogenetic tree for the MNSA using MEGA v7.0.18 [35] software and utilized FigTree v1.4.2 (http://tree.bio.ed.ac.uk/software/figtree/) to visualize the tree.

### 2.7. Tests for Selection Signals

To investigate the selection signals for SGCSSs, we performed the nucleotide diversity and Tajima’s D statistics for each of the five species (sheep, goat, cow, pig, and chicken) using the 1450 WGS individuals described above. All data processing was carried out using Plink v1.9 and the VCFtools v0.1.13 software [36,37]. For each species population, the nucleotide diversity and Tajima’s D were calculated applying a sliding window strategy with window of 1000 bp and shifting step size of 250 bp by using the scikit-allel v0.20.3 package (https://github.com/cggh/scikit-allel).

## 3. Results and Discussion

### 3.1. Identification of the Highly Confident SGCSSs

**Figure 2:**
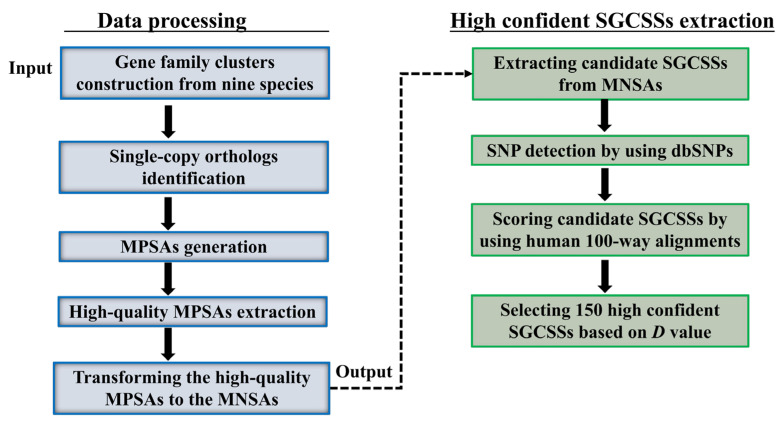
Overview of the highly confident SGCSSs identification. MPSA, multiple peptide sequence alignment; MNSA, multiple nucleotide sequence alignment.

**Figure 3:**
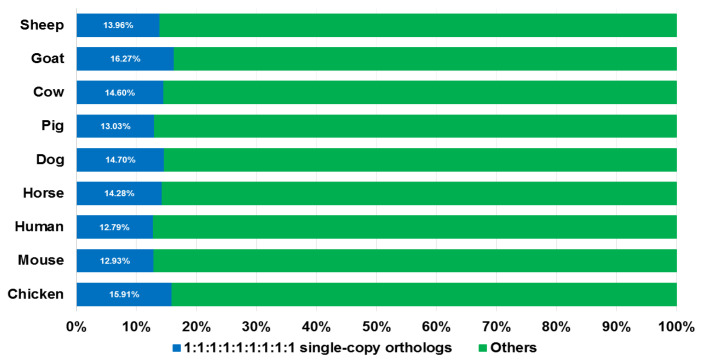
Single copy orthologs examined in the nine species. The proportion of single copy genes in whole genes of every species are represented in blue color and other types are shown in green.

The overall process for searching SGCSSs is shown in Figure 2. We started with inferring the orthologs among nine species (sheep, goat, cow, pig, dog, horse, human, mouse, and chicken) using TreeFam. As a result, a total of 2916 single copy gene families were identified from 12,361 orthologous groups, with an average of 14.27% in nine genomes (ranging from 12.79% in human to 16.27% in goat, Figure 3). After translating the CDS to peptide sequences, we performed multiple sequence alignments for each single copy ortholog. Moreover, 92% of the length of MPSAs exceeded 100 amino acids, indicating high-quality assemblies and annotations for the nine investigated genomes. However, there were only 810 (27.7%) MPSAs with a length longer than 100 amino acids and proportion of gaps less than 20%. This might be explained by the reason that the nine species were evolutionarily distant.

To obtain highly confident SGCSSs, as shown in Table 1, we applied a serial of filtering steps, starting with 3245 candidate SGCSSs from 810 MNSAs. We then found that 3230 out of 3245 candidate SGCSSs, along with their corresponding sites among the other seven species, had exact genomic coordinates. After that, the sites that had SNP records in the dbSNP data were filtered, resulting in 2136 candidate SGCSSs.

These candidate SGCSSs were only identified from nine species, but it is still unclear whether these sheep and goat specific sites exist in other species. Therefore, we downloaded the human 100-way alignment data, which contain the alignment information for 100 species from UCSC, to filter out sites that were not specific to sheep and goat. After filtering out non-recorded sites and performing quality control for missing data, the number of candidates were reduced to 1158. Meanwhile, the species in which the frequency of missing sites exceeded 10% during the quality control step were discarded, and 96 species remained. Finally, the top-ranked 150 sites based on the *D* value were selected as the SGCSSs list (Supplementary Table S4).

An ideal SGCSS should be in accordance with the rules of low intraspecific heterogeneity and high interspecific divergence. In this study, low intraspecific heterogeneity was defined as follows: the allele at the SGCSS locus, where the base was identical to the base (or its complementary base) on the sheep reference genome, must have exhibited a very high frequency (>0.95), whereas another allele, whose base differed from the sheep reference genome should have had a sharply low frequency (<0.05). High interspecific divergence was defined as follows: these SGCSSs could be clearly separated from other species using some clustering algorithms, such as PCA. Therefore, we next conducted a performance evaluation on the 150 SGCSSs based on these two principles.

### 3.2. Evaluation of Intraspecific Heterogeneity

Our first step was to verify whether the 150 SGCSSs adhered to the principle of low intraspecific heterogeneity. The resequencing data containing 1450 animals from five species were used to carry out this test. We calculated the frequency of the site that was homozygous and consistent with the reference genome sequence (homozygous reference genotype). We found high intraspecific consistency for these 150 SGCSSs. As shown in Table 2, only 4 (2.7%) out of the 150 SGCSSs exhibited allele variation in certain species. Nevertheless, we found an extremely high proportion of homozygous reference genotypes in all of four SGCSSs (0.950–1). This suggests that the final SGCSSs we selected were highly reliable.

### 3.3. Evaluation of Interspecific Divergence

**Figure 4:**
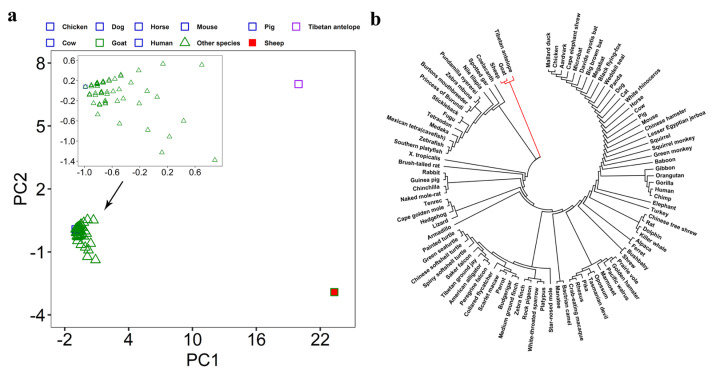
The PCA and phylogenetic analysis among 96 species by using 150 SGCSSs and their corresponding sites from the other 94 species. (**a**) First two principal components are used for these 96 species. (**b**) The unrooted phylogenetic tree constructed using maximum-likelihood algorithm in MEGA v7.0.18. The lines in red color refer to sheep, goat, and Tibetan antelope.

Our second step was to verify whether these SGCSSs exhibited high interspecific divergence. We performed PCA on these 150 SGCSSs and their corresponding sites in the other 94 species, which were extracted from the human 100-way alignment data after quality control and converted from the ACGT format to integers. As expected, the result clearly exhibited separation between sheep–goat and the other 94 species (Figure 4a). It is worth noting that the PCA result also revealed that the Tibetan antelope, which is a member of the same *Caprinae* subfamily as sheep and goat, could be clearly distinguished from other species. Although these SGCSSs were screened from the datasets without genomic information about Tibetan antelope, they clustered closer with sheep and goat rather than other species. This indicated that these SGCSSs could also be used for species identification between Tibetan antelope and sheep–goat. Phylogenetic analysis provided a consistent result. As shown in Figure 4b, the maximum-likelihood phylogenetic tree revealed that sheep, goat, and Tibetan antelope were grouped more closely together than other species. Interestingly, we also observed that sheep, goat, and Tibetan antelope clustered closely to many aquatic animals, such as spotted gar, zebrafish, and southern platy fish, in the phylogenetic tree. This thus implied that these 150 SGCSSs might have appeared very early in evolution.

Overall, our analysis showed that these 150 SGCSSs meet the principles of low intraspecific heterogeneity and high interspecific divergence.

### 3.4. Characterization of Selected SGCSSs

We then analyzed the genes containing these SGCSSs using sheep genomic coordinates. There were 103 genes with these sites. Most genes contained only one SGCSS, while a few contained more than one, such as *UBR4* (n = 9), *NCOA6* (n = 6), *HELZ* (n = 5), *TRIP12* (n = 4), and *BIRC6* (n = 4) (Supplementary Table S4).

**Figure 5:**
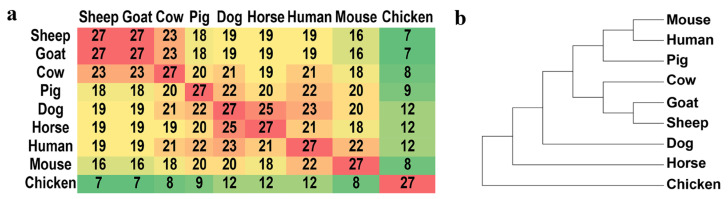
The matrix for the number of common non-synonymous substitutions between nine species (**a**) and the phylogenetic tree constructed using 27 non-synonymous sites in nine species (**b**).

We defined an SGCSS as a non-synonymous site if amino acid alternations occurred in any of the other seven species or a synonymous site if no amino acid alternation was observed in either of the other seven species. (details in Supplementary Table S5). It was found that 123 (82%) sites were synonymous, while the remaining 27 (18%) sites were non-synonymous substitutions. Among the 27 non-synonymous sites, cow shared the highest number of identical amino acids with sheep and goat (n = 23), while chicken shared the lowest number (n = 7) (Figure 5a). Considering the evolutionary distance among the nine species, it was implied that 27 non-synonymous substitutions had the power to effectively distinguish these nine species (Figure 5b).

### 3.5. Case Study for Functionality of SGCSSs

**Figure 6:**
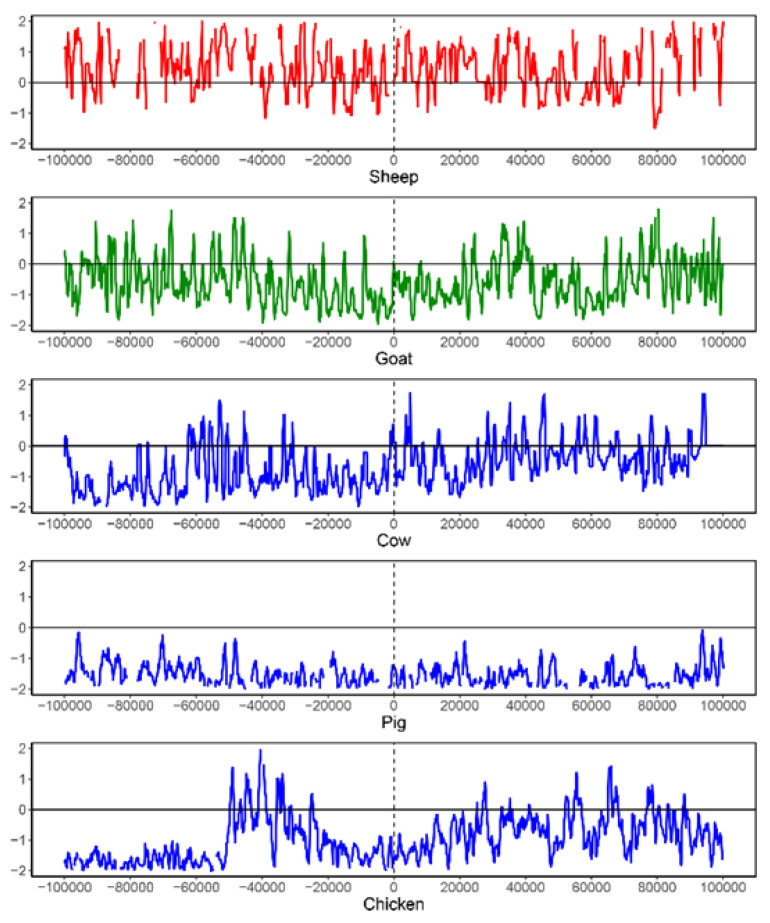
The Tajima’s D analysis for an SGCSS and its corresponding sites on five species. The SGCSS is located at chr13:63638708 of sheep reference genome. To perform this analysis, a 200 kb region centered on the SGCSS is extracted from sheep genome (red). Similarly, the 200 kb regions centered on the corresponding sites are extracted from goat (green), cow (blue), pig (blue), and chicken (blue) reference genomes.

As a case study, we observed that the SGCSSs located on the *NCOA6* gene exhibited a high level of differentiation between sheep–goat and the other seven species (Supplementary Table S5). As a nuclear receptor coactivator, *NCOA6* plays an essential role in many biological functions, such as the regulation of transcription, pigmentation, adipocyte differentiation, etc. [38,39,40,41]. *NCOA6* is located on sheep chromosome 13, and two non-synonymous substitutions associated with sheep and goat common specific peptides (SGCSPs) were detected from the 150 SGCSSs (Supplementary Table S5). The sites for these two peptides were located at chr13:63638684 and chr13:63638708 of the sheep reference genome. We further detected the selection signals by using 1450 resequencing individuals from five species. Since the distance between these two sites was very close (24 bp), 200 kb regions centered at chr 13:63638708 of the sheep reference genome or its corresponding sites on goat, cow, pig, and chicken reference genomes were respectively selected. Low levels of nucleotide diversity were observed in all species (Supplementary Figure S2 and Supplementary Table S6), especially near chr 13:63638708 or its corresponding sites. Negative Tajima’s D values, indicating positive or negative selection, were also observed in the goat, cow, pig, and chicken populations, particularly in the left regions of the SGCSS site (Figure 6 and Supplementary Table S6). Thus, the analysis of nucleotide diversity and Tajima’s D indicated that the regions near these two SGCSSs or its corresponding sites might have experienced natural selection in five species.

## 4. Conclusions

In conclusion, we built a pipeline and identified 150 SGCSSs through extensive genome scanning. The SGCSSs effectively distinguished sheep and goat from the other seven species with both low intraspecific heterogeneity and high interspecific divergence. Our results showed that species identification could be obtained from nuclear genomes, such as these SGCSSs, which was an efficient and cost-effective strategy. Since these SGCSSs were selected from single copy genes, this thus provided the potential for quantitative analysis, although this was not included in the current study. The existence of non-synonymous SGCSSs could be due to genetic drift or functional alternation. Our approach also provided an example for an SGCSS of selection signal identification in the genome.

## Figures and Tables

**Table 1:** The number of remaining candidate SGCSSs after each filter step.

Before	After	Filter Factors
-	3245	Non-candidate SGCSSs
3245	3230	No genomic coordinates
3230	2136	SNPs observed in dpSNP
2136	1158	No records or quality control (human 100-way alignments)
1158	150	*D* value

**Table 2:** The frequency of homozygous reference genotypes of 4 sites of 150 SGCSSs. The coordinates of these four sites are based on sheep reference genome.

Chr	Position	Genotype1	Genotype2	Sheep	Goat	Cow	Pig	Chicken
5	47181590	GG, CC	AA, TT	1	1	1	0.984	1
12	10283861	TT	GG, CC	1	1	1	1	0.993
12	40543138	AA	GG, CC	1	1	1	1	0.993
21	39262113	CC	AA, TT	0.95	1	1	1	1

Genotype1 represents the homozygous reference genotype in sheep and goat. Genotype2 represents the homozygous reference genotype in cow, pig, and chicken.

## Data Availability

The original contributions presented in the study are included in the article.

## Supplementary Materials

The following supporting information can be downloaded at: https://www.mdpi.com/article/10.3390/biom14060638/s1, Supplementary Table S1. The reference genome versions used for identifying candidate SGCSSs. Supplementary Table S2. Detailed information of 1450 resequencing samples. Supplementary Table S3. The number of SNP recorded in Ensembl dbSNP database (version 92). Supplementary Table S4. Summary of 150 SGCSSs and their corresponding sites among the other seven species. The position for each SGCSS is based on sheep reference genome. The nucleobases are extracted from nine species reference genomes. Supplementary Table S5. The substitution types of 150 SGCSSs. The position for each SGCSS is based on sheep reference genome. The nucleobases are extracted from nine species CDS data and then translated to peptides. Supplementary Table S6. The nucleotide diversity and Tajimas’D analysis for an SGCSS and its corresponding sites on five species. Supplementary Figure S1. A schematic diagram showing a group of probes in a species. Supplementary Figure S2. The nucleotide diversity analysis for an SGCSS and its corresponding sites on five species.
