# Supplementary material for: Genome-Wide Identification of Specific Genetic Loci Common to Sheep and Goat
Source: Biomolecules. 2024 May 29;14(6):638. doi: 10.3390/biom14060638 (PMC11201639; doi:10.3390/biom14060638)
Supplement: Supplementary file 1 [file biomolecules-14-00638-s001.zip › biomolecules-supplementary figure.pdf]

biomolecules

*Supplementary Materials*

# Genome-Wide Identification of Specific Genetic Loci Common to Sheep and Goat

**Zuoxiang Liang [1,2,†], Xiaoyu Yue [1,†], Yangxiu Liu [1], Mengyan Ye [1], Ling Zhong [1], Yue Luan [1] and Qin Wang [1,*]**

1 State Key Laboratory of Animal Biotech Breeding, National Engineering Laboratory for Animal Breeding, Laboratory of Animal Genetics, Breeding and Reproduction, Ministry of Agriculture and Rural Affairs, College of Animal Science and Technology, China Agricultural University, Beijing 100193, China; zliang@umn.edu (Z.L.); b20233040387@cau.edu.cn (X.Y.); 18813039611@163.com (Y.L.); mengyanye@cau.edu.cn (M.Y.); sy20213040767@cau.edu.cn (L.Z.); luanyue@cau.edu.cn (Y.L.)
2 Department of Animal Science, University of Minnesota, Saint Paul, MN 55108, USA
* Correspondence: wangqin@cau.edu.cn
† These authors contributed equally to this work.

## Supplementary Figures

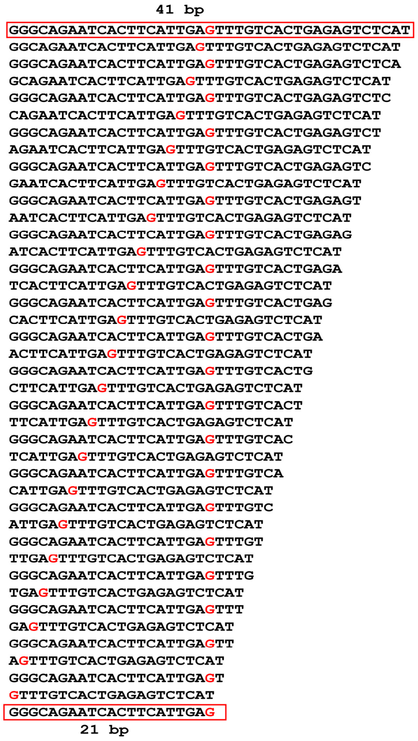


**Supplementary Figure S1.** A schematic diagram showing a group of probes in a species. The sequence is first extracted from the MNSA window, and then trimmed into 40 probes. A group of probes consists of a raw sequence with the length of 41 bp (red rectangle) and 40 probes with the minimum length of 21 bp (red rectangle). They all contain the candidate SGCSS (red).

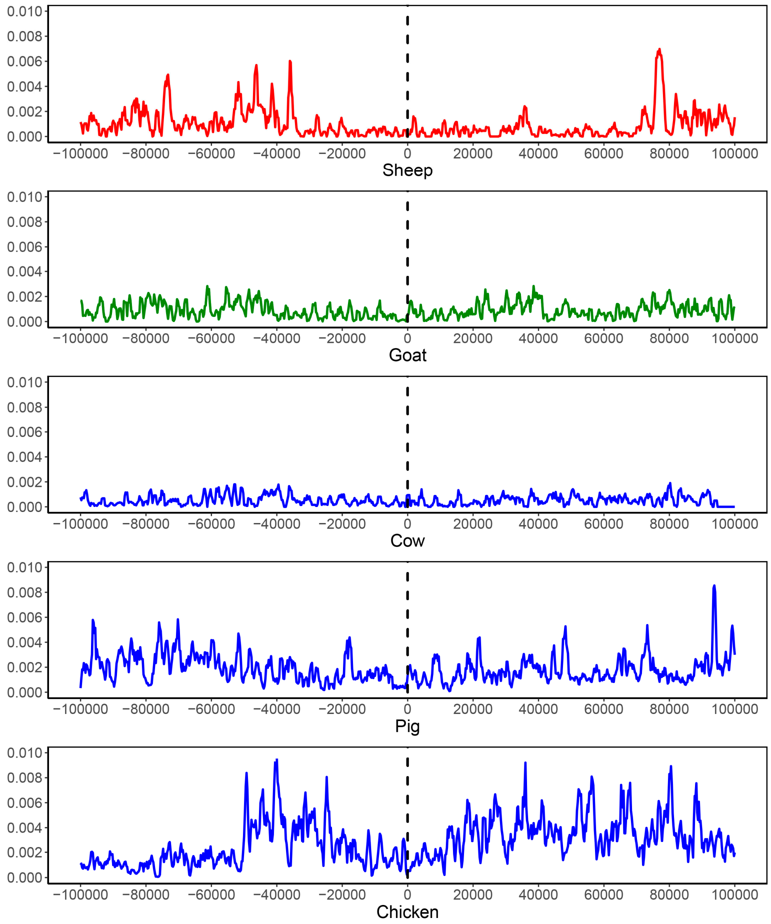


**Supplementary Figure S2.** The nucleotide diversity analysis for an SGCSS and its corresponding sites on five species. The SGCSS is located at chr13:63638708 of sheep reference genome. To perform this analysis, a 200 kb region centered on the SGCSS is extracted from sheep genome (red). Similarly, the 200 kb regions centered on the corresponding sites are extracted from goat (green), cow (blue), pig (blue) and chicken (blue) reference genomes.
